# Supplementary material for: The Importance of a Distance between the Lines Encircling Pulmonary Veins in Atrial Fibrillation Ablation on First-Pass Isolation Ratio and Clinical Outcomes
Source: Int J Environ Res Public Health. 2023 Mar 24;20(7):5250. doi: 10.3390/ijerph20075250 (PMC10094726; doi:10.3390/ijerph20075250)
Supplement: Supplementary file 1 [file ijerph-20-05250-s001.zip › ijerph-2206831-supplementary.pdf]

Table S1. Logistic regression analysis for recurrence of atrial arrhythmia - univariate analysis.

	Odds ratio (95% CI)	P-value
Age	0.99 (0.96-1.04)	0.95
Gender	0.53 (0.21-1.32)	0.17
BMI	0.99 (0.89-1.09)	0.80
Arterial hypertension	1.04 (0.41-2.66)	0.94
Diabetes mellitus	0.98 (0.37-2.63)	0.97
Coronary artery disease	2.57 (0.98-6.68)	0.054
Heart failure	1.90 (0.50-7.25)	0.35
Ejection fraction	0.95 (0.89-0.998)	0.043
The upper distance between lines (mm)	0.99 (0.94-1.04)	0.71
The lower distance between lines (mm)	1.01 (0.97-1.05)	0.64
The area between the lines indexed to left atrial diameter	0.81 (0.42-1.54)	0.52

Table S2. Logistic regression analysis for recurrence of atrial arrhythmia- multivariate analysis.

	Odds ratio (95% CI)	P-value
Age	0.99 (0.93-1.05)	0.62
Gender	1.21 (0.38-3,80)	0.75
Coronary artery disease	3.58 (0.99-12.90)	0.052
Ejection fraction	0.95 (0.88-1.02)	0.13
The upper distance between lines (mm)	0.996 (0.93-1.07)	0.92
The lower distance between lines (mm)	0.991 (0.92-1.07)	0.83
The area between the lines (cm2)	1.09 (0.72-1.65)	0.68
The area between the lines indexed to left atrial diameter	0.45 (0.07-2.70)	0.38
